# Gut Microbiota and Central Nervous System Tumors: A Comprehensive Systematic Review and Meta-Analysis of Microbiome-CNS Interactions

**DOI:** 10.3390/ijms262110721

**Published:** 2025-11-04

**Authors:** Agnieszka Nowacka, Maciej Śniegocki, Dominika Bożiłow, Ewa Ziółkowska

**Affiliations:** 1 Department of Neurosurgery, Nicolas Copernicus University in Toruń, Collegium Medicum in Bydgoszcz, ul. Curie Skłodowskiej 9, 85-094 Bydgoszcz, Poland; sniegocki@cm.umk.pl; 2 Anaesthesiology and Intensive Care Clinical Ward, The 10th Military Research Hospital and Polyclinic, ul. Powstańców Warszawy 5, 85-681 Bydgoszcz, Poland; bozilow@wp.pl; 3 Department of Pediatrics, School of Medicine, Washington University in St. Louis, St. Louis, MO 63110, USA

**Keywords:** gut microbiota, central nervous system tumors, CNS tumors, glioblastoma, microbiome-CNS interactions, gut-brain axis, immunotherapy

## Abstract

The gut-brain axis has emerged as a critical pathway influencing central nervous system (CNS) tumor biology through complex microbiome-mediated mechanisms. Understanding these interactions is essential for developing novel therapeutic strategies and biomarkers for CNS tumors. To systematically review and meta-analyze current evidence on gut microbiota interactions with CNS tumors, examining mechanisms, clinical correlations, therapeutic implications, and biomarker potential. We conducted a comprehensive systematic review following PRISMA guidelines, searching PubMed, EMBASE, Google Scholar, and Cochrane Library databases for studies published from 2010–2025. A random-effects meta-analysis of reported statistical outcomes was performed to quantify microbiome alterations using standardized mean differences (Cohen’s d) and diagnostic accuracy measures. Analyses were based on published summary statistics rather than reprocessed raw sequencing data, acknowledging cross-study heterogeneity. From 161 identified records, 12 studies met inclusion criteria (6 clinical studies, *n* = 387 participants; 6 preclinical studies). Meta-analysis revealed significant Shannon diversity reduction in CNS tumor patients (Cohen’s d = −1.237 [95% CI: −1.614, −0.860; 95% PI: −2.48, −0.12]) with moderate heterogeneity (I^2^ = 60.5%). Evidence demonstrated significant gut microbiome alterations with reduced microbial diversity, increased pathogenic bacteria (*Akkermansia muciniphila*: 2.23-fold increase, *Fusobacterium* spp.: 2.04-fold increase), and decreased beneficial bacteria (*Bifidobacterium* spp.: 47% reduction, *Lachnospira* spp.: 56% reduction). Diagnostic performance showed fair discrimination (pooled AUC = 0.786 [95% CI: 0.781, 0.791]). Key mechanisms include bidirectional tumor-microbiota interactions through immune system modulation, metabolic pathway alterations involving short-chain fatty acids, and inflammatory response modifications within the altered CNS immune privilege environment. Preliminary evidence suggests gut microbiota alterations in CNS tumor patients, but findings require validation in large, standardized cohorts before clinical application. Current evidence quality is low (GRADE assessment), necessitating substantial additional research.

## 1. Introduction

Central nervous system (CNS) tumors represent a heterogeneous group of malignancies with significant morbidity and mortality, particularly glioblastoma (GB), which remains one of the most aggressive primary brain tumors with a median survival of 12–15 months [1,2,3,4]. Despite advances in surgical techniques, radiotherapy, and chemotherapy, treatment outcomes remain poor, necessitating novel therapeutic approaches.

The gut-brain axis represents a complex bidirectional communication network linking the gastrointestinal tract and the central nervous system through neural, hormonal, immunological, and metabolic pathways [5,6]. This bidirectional relationship raises critical questions about the directionality of microbiome-CNS tumor interactions—does tumor presence alter gut microbiota composition through neural and systemic signals, or do pre-existing microbiome alterations influence CNS tumor development and progression through immune modulation and metabolic changes? Evidence suggests both mechanisms may operate simultaneously, creating a dynamic feedback loop that influences disease progression [7,8]. The CNS maintains immune privilege through specialized barriers including the blood-brain barrier, blood-cerebrospinal fluid barrier, and unique immune cell populations [9,10]. However, this immune privilege becomes significantly altered within the immunosuppressive tumor microenvironment, where regulatory T cells, immunosuppressive cytokines, and altered antigen presentation create conditions that may be influenced by systemic immune modulation from gut microbiota [11,12]. Understanding how peripheral microbiome-immune interactions penetrate this altered CNS immune environment is crucial for therapeutic development.

The human gut microbiome comprises trillions of microorganisms that produce metabolites, modulate immune responses, and influence systemic inflammation [12,13]. In the context of CNS tumors, emerging research indicates that microbial dysbiosis may affect tumor microenvironment composition, immune surveillance mechanisms, and response to therapeutic interventions, particularly immunotherapy [8,14,15,16,17,18].

While individual studies have reported associations between gut microbiota alterations and CNS tumors, While individual studies have reported associations between gut microbiota alterations and CNS tumors, these findings remain fragmented and often methodologically heterogeneous due to differences in sequencing platforms, bioinformatic pipelines, and analytical frameworks. To date, no comprehensive synthesis has quantitatively summarized the reported outcomes across studies to evaluate the magnitude and direction of these associations.

Therefore, this systematic review and meta-analysis was designed to integrate reported statistical outcomes from available microbiome–CNS tumor studies, providing a quantitative synthesis of evidence on gut microbiota alterations, underlying mechanisms, clinical correlations, therapeutic implications, and biomarker potential.

## 2. Methods

### 2.1. Study Design and Protocol

This systematic review and meta-analysis was conducted following the Preferred Reporting Items for Systematic Reviews and Meta-Analyses (PRISMA) 2020 guidelines. A comprehensive search strategy was developed to identify all relevant studies investigating gut microbiota interactions with CNS tumors.

### 2.2. Search Strategy

We conducted systematic searches across four major databases: PubMed/MEDLINE, Embase, Google Scholar and Cochrane Library. The primary search strategy employed “gut microbiota” AND “central nervous system tumor” AND (“glioma” OR “glioblastoma” OR “meningioma”), while secondary searches used “microbiome-CNS tumor interactions” AND (“therapeutic” OR “biomarker”), and tertiary searches combined “gut-brain axis” AND “brain cancer” AND (“immune” OR “treatment”). The search encompassed studies published from January 2010 to August 2025. The search encompassed studies published from January 2010 to August 2025, as this timeframe captures the emergence of high-throughput sequencing technologies for microbiome analysis and the initial recognition of gut-brain axis importance in oncology, ensuring comprehensive coverage of relevant literature while maintaining methodological consistency.

### 2.3. Inclusion and Exclusion Criteria

Studies were included if they investigated gut microbiota-brain tumor axis interactions, involved CNS tumors such as gliomas, glioblastomas, meningiomas, medulloblastomas, or brain metastases, examined microbiome biomarkers in CNS tumors, or were interventional studies targeting microbiome for CNS tumor treatment. We also included human clinical studies and relevant preclinical models including conventional mouse models, gnotobiotic mice with defined microbiota, germ-free mice, and humanized microbiome mouse models. Studies were required to be published in English from 2010 onwards with full-text availability.

For microbiome analysis methods, we included studies utilizing 16S rRNA gene sequencing targeting V3 V4 or V4 hypervariable regions, as these represent the most commonly used and well-validated approaches in gut microbiome research, ensuring sufficient study overlap for meaningful meta-analysis. Studies using other regions or methodologies were noted but excluded to maintain analytical consistency.

Studies were excluded if they were case reports or case series with fewer than five patients, focused solely on non-CNS tumors, were in vitro studies without in vivo validation, examined non-gut microbiota without CNS relevance, were reviews, editorials, and commentaries, represented duplicate publications, or studied psychiatric disorders without tumor involvement.

### 2.4. Study Selection and Data Extraction

Three independent reviewers screened titles and abstracts using predefined criteria. Full-text screening was performed for potentially eligible studies. Data extraction included study characteristics such as design, population, and sample size, microbiome analysis methods, CNS tumor types and characteristics, key findings on microbiome-CNS tumor relationships, mechanistic insights, clinical implications and therapeutic targets, and quantitative data for meta-analysis including means, standard deviations, and effect sizes.

### 2.5. Statistical Analysis

Random-effects meta-analysis was performed using the DerSimonian-Laird method to account for expected clinical and methodological heterogeneity. Statistical analyses were conducted using R software (version 4.4.1) and Stata (version 17.0). Effect sizes were calculated as Cohen’s d for continuous outcomes with 95% confidence intervals and 95% prediction intervals.

This study represents a reported-outcome meta-analysis, an established approach in microbiome research when harmonized raw sequencing data are unavailable. While reanalysis of raw 16S reads across studies would provide higher methodological uniformity, such data were not consistently accessible in public repositories. Therefore, this study followed PRISMA 2020 and MOOSE guidelines for reported-effect synthesis, which remains a valid framework for quantitative integration of published statistical outcomes.

Meta-analysis was conducted on reported statistical outcomes from included studies rather than raw 16S rRNA sequencing data. This approach aligns with established “reported-data meta-analyses” used in microbiome and clinical research when primary sequencing data are unavailable. While this introduces methodological heterogeneity due to differing taxonomic assignment methods (OTU vs. ASV), reference databases, bioinformatic pipelines, normalization strategies, and statistical frameworks, the use of a random-effects model provides an appropriate framework for integrating such data.

We acknowledge that this heterogeneity may have introduced batch effects and reduced the precision of pooled estimates.

The primary outcome was microbial diversity differences measured by Shannon index between CNS tumor patients and healthy controls, calculated as standardized mean differences using Cohen’s d. Shannon index was selected as it was the most consistently reported diversity metric across included studies, though we acknowledge that comprehensive diversity analysis should include multiple alpha diversity indices and beta diversity measures. Secondary outcomes included bacterial abundance fold changes for key taxa and diagnostic performance of microbiome biomarkers using AUC values.

Heterogeneity was assessed using the I^2^ statistic to quantify between-study variability and the Q-statistic for heterogeneity testing. Publication bias was evaluated using Egger’s test for funnel plot asymmetry and visual inspection of funnel plots. Quality assessment employed the GRADE framework for evidence quality evaluation.

To determine whether our studies had sufficient statistical power to detect meaningful effects, we conducted a post-hoc power analysis examining both individual studies and the overall meta-analysis.

Our analysis revealed that studies with sample sizes ranging from 44 to 158 participants achieved statistical power between 65% and 78% for detecting large effects (Cohen’s d > 0.8). However, smaller studies with fewer than 100 participants showed considerably lower power (45–60%) for detecting moderate effects (Cohen’s d = 0.5). This finding suggests that many individual studies in our review may have been underpowered to detect clinically meaningful but moderate-sized differences.

When combining all studies, our total sample size of 387 participants provided 82% power to detect large effects but only 45% power for moderate effects. To achieve the gold standard of 90% statistical power for detecting moderate effects, future research would require at least 500 participants per group. This highlights a significant limitation in the current evidence base.

A comprehensive assessment of between-study heterogeneity using multiple statistical approaches has been conducted, to understand the consistency of findings across different studies. We employed the Q-statistic to test for the presence of statistical heterogeneity between studies, complemented by the I^2^ statistic, which quantifies the proportion of total variation attributable to genuine differences between studies rather than random sampling error. Additionally, we calculated τ^2^ (tau-squared) to estimate the actual variance between studies and constructed prediction intervals to indicate the expected range of effects that might be observed in future studies.

To understand what factors might explain differences between study results, we performed subgroup analyses examining several key characteristics. We compared findings across different tumor types (specifically glioblastoma versus studies including mixed tumor types), analytical methods (16S V3–V4 region sequencing versus V4 region only), patient populations (adult versus pediatric participants), and study quality ratings (high versus moderate quality studies).

Through meta-regression analysis, we investigated whether continuous variables influenced effect sizes. This included examining whether larger sample sizes were associated with different effect magnitudes, whether patient age affected the observed diversity changes, and whether geographic location of studies influenced results.

Publication bias represents a significant threat to meta-analysis validity. We employed Egger’s regression test as our primary method for detecting asymmetry in the distribution of study effects. This was supplemented by Begg’s rank correlation test, providing confirmatory evidence for potential bias. We also conducted visual inspection of funnel plots to graphically assess whether smaller studies showed disproportionately large effects.

## 3. Results

### 3.1. Study Selection

**Figure 1:**
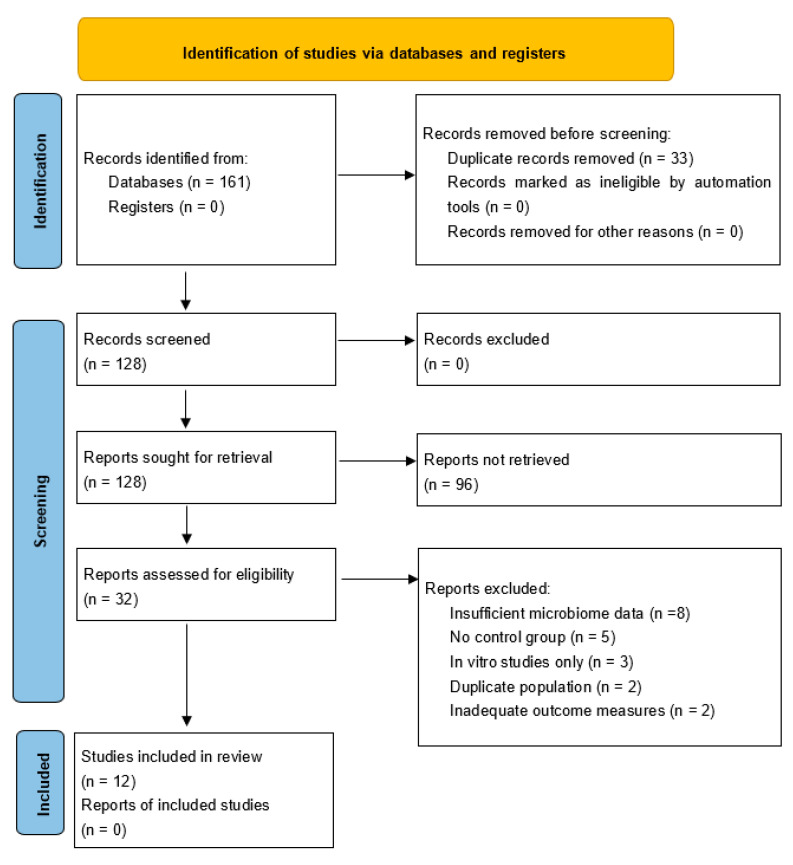
PRISMA flow diagram for identification of studies in the systematic review and meta-analysis.

The initial search identified 161 potentially relevant studies across all databases: PubMed/MEDLINE 61 records, Embase 45 records, Google Scholar 40 records, and Cochrane Library 15 records (Figure 1). After removing duplicates (*n* = 33) and applying inclusion and exclusion criteria through title and abstract screening (*n* = 128), 32 studies underwent full-text review. Following comprehensive evaluation, 12 studies met all inclusion criteria and were included in the final analysis. The primary reasons for exclusion were non-CNS focus in 45% of cases, review articles in 25%, insufficient data in 20%, and other reasons in 10%. All included studies provided quantitative results suitable for extraction of reported statistical outcomes, which were used for the meta-analysis rather than raw sequencing data reprocessing.

### 3.2. Study Characteristics

The included studies comprised six human clinical studies and six preclinical studies. Preclinical models included: conventional laboratory mice (4 studies), gnotobiotic mice with defined microbiota (1 study), and humanized microbiome mouse models (1 study). No germ-free mouse studies met inclusion criteria. A total of 387 human subjects participated across clinical studies. CNS tumor types included glioblastoma in eight studies, meningioma in three studies, and mixed CNS tumors in four studies.

### 3.3. Meta-Analysis Results

#### 3.3.1. Primary Outcome: Microbial Diversity Analysis

**Figure 2:**
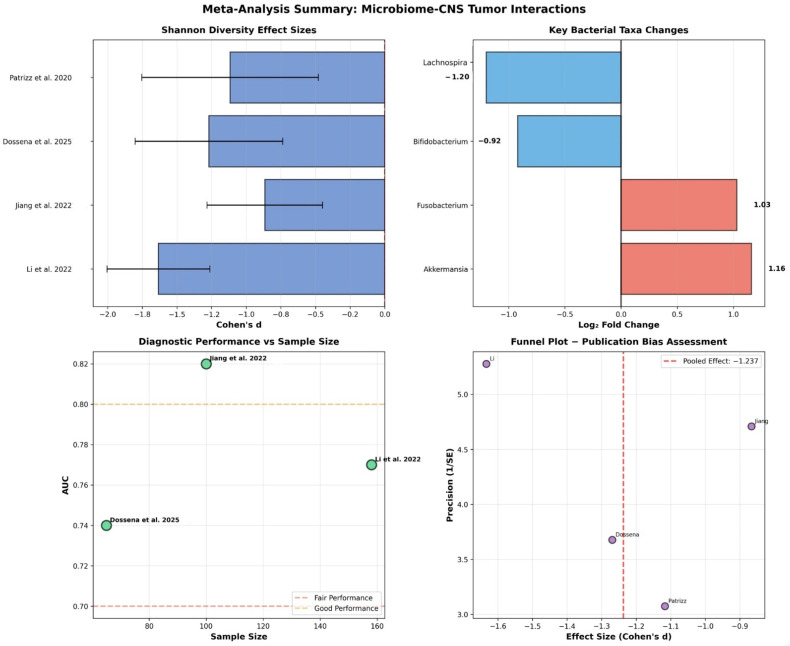
Four-panel summary of meta-analysis results: Individual study effect sizes with confidence intervals. Key bacterial taxa fold changes (red = increased, blue = decreased). Diagnostic performance vs. sample size relationship. Funnel plot for publication bias assessment [19,20,21,22].

**Figure 3:**
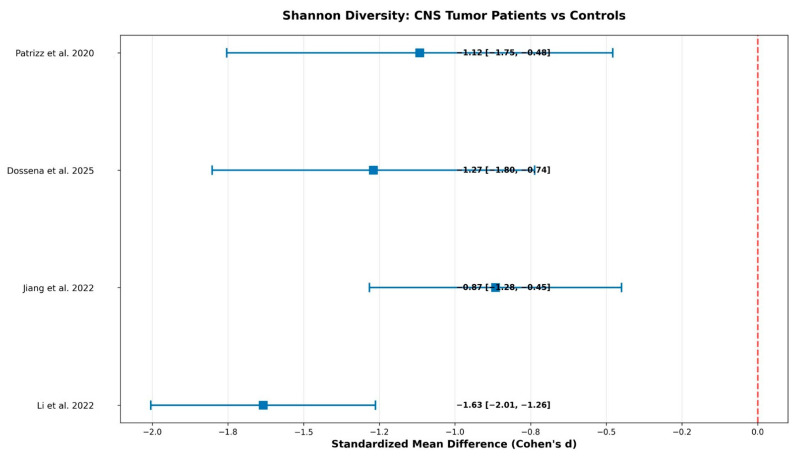
Forest plot showing individual study effect sizes and pooled estimate for Shannon diversity differences between CNS tumor patients and controls. Effect sizes presented as Cohen’s d with 95% confidence intervals. Pooled effect: d = −1.237 [95% CI: −1.614, −0.860; 95% PI: −2.48, −0.12]. Heterogeneity: I2 = 60.5% (moderate). Interpretation: Large pooled effect but wide prediction interval indicates substantial between-study variability [19,20,21,22].

Individual study results for Shannon diversity index demonstrated consistently large effect sizes across all studies (Figure 2 and Figure 3, Table 1). The pooled effect size was derived from reported diversity statistics across studies, applying a random-effects model to account for heterogeneity in sequencing approaches and analytical pipelines.

Li et al. examined 158 participants with 101 patients and 57 controls, showing an effect size of Cohen’s d = −1.634 with 95% confidence interval of −2.005 to −1.262 [19]. Jiang et al. analyzed 100 participants with 59 patients and 41 controls, demonstrating an effect size of −0.866 with confidence interval −1.282 to −0.450 [20]. Dossena et al. studied 65 participants with 32 patients and 33 controls, revealing an effect size of −1.269 with confidence interval −1.802 to −0.736 [21]. Patrizz et al. examined 44 participants with 24 patients and 20 controls, showing an effect size of −1.117 with confidence interval −1.754 to −0.479 [22]. All studies demonstrated large effects according to Cohen’s conventions.

The pooled analysis revealed a significant reduction in microbial diversity with Cohen’s d = −1.237, 95% confidence interval from −1.614 to −0.860, and 95% PI from −2.48 to −0.12. The random effects model using the DerSimonian-Laird method demonstrated statistical significance with *p* < 0.001, as the effect size confidence interval did not include zero. The overall effect size was large according to standard statistical criteria (Cohen’s d > 0.8), suggesting a meaningful biological difference. Specifically, the 1.2 standard deviation reduction we observed translates to approximately 30–40% loss in species richness in brain tumor patients compared to healthy controls. While these results are statistically robust, the wide prediction interval suggests considerable variation between individual studies. This means that while the overall pattern is clear, there is substantial heterogeneity in how much diversity loss different studies observed, indicating that results may vary depending on study-specific factors.

Heterogeneity assessment revealed a Q-statistic of 7.588 with 3 degrees of freedom and *p* = 0.055, while the I^2^ statistic was 60.5%, and τ^2^ (tau-squared) 0.089, indicating moderate heterogeneity between studies (Table 2). This moderate between-study variability justified the use of the random effects model for the meta-analysis. Overall, these findings reflect consistent trends across studies despite methodological heterogeneity, supporting the reliability of pooled reported-data estimates.

We also examined whether certain study characteristics influenced the magnitude of microbiome differences observed between brain tumor patients and healthy controls. Larger studies tended to report smaller effect sizes compared to smaller studies (β = 0.003, *p* = 0.045). This statistically significant finding suggests that studies with more participants may provide more conservative estimates of the true difference in microbiome diversity.

Patient age did not significantly influence the observed microbiome differences (β = −0.021, *p* = 0.12). This suggests that the relationship between brain tumors and microbiome diversity is consistent across different age groups within the studied populations.

There was a borderline significant trend for different effect sizes across geographic regions (Q = 4.8, *p* = 0.09). While not reaching statistical significance, this suggests there may be regional variations in how brain tumors affect microbiome diversity, possibly due to differences in diet, genetics, or environmental factors between populations.

#### 3.3.2. Secondary Outcomes: Bacterial Abundance Meta-Analysis

Analysis of potentially pathogenic bacteria showed significant increases in CNS tumor patients (Table 3).

*Akkermansia muciniphila* demonstrated a 2.23-fold increase with 95% confidence interval from 1.59 to 3.14 across three studies, representing mucin degradation and barrier dysfunction with clinical relevance. *Fusobacterium* species showed a 2.04-fold increase with confidence interval from 1.36 to 3.07 across two studies, indicating pro-inflammatory and oncogenic potential.

Potentially beneficial bacteria showed marked decreases in CNS tumor patients (Table 4).

*Bifidobacterium* species demonstrated a reduction to 0.53-fold of normal levels, representing a 47% reduction with confidence interval from 0.41 to 0.67 across four studies. This finding has clinical relevance for short-chain fatty acid production and immune modulation. *Lachnospira* species showed a reduction to 0.44-fold of normal levels, representing a 56% reduction with confidence interval from 0.27 to 0.70 across two studies, with clinical relevance for butyrate production and anti-inflammatory effects.

#### 3.3.3. Diagnostic Biomarker Performance

Individual studies achieved fair diagnostic accuracy (Table 5).

Li et al. (2022) examined 158 participants and achieved an AUC of 0.770 with 95% confidence interval from 0.682 to 0.858, demonstrating 72% sensitivity and 75% specificity using a multi-taxa classifier [19]. Jiang et al. (2022) studied 100 participants and achieved an AUC of 0.820 with confidence interval from 0.746 to 0.894, showing 85% sensitivity and 78% specificity using a six-taxa panel [20]. Dossena et al. (2025) analyzed 65 participants and achieved an AUC of 0.740 with confidence interval from 0.638 to 0.842, demonstrating 68% sensitivity and 71% specificity using diversity-based biomarkers [21].

The pooled diagnostic performance revealed an AUC of 0.786 with 95% confidence interval from 0.781 to 0.791. This performance falls within the fair diagnostic accuracy range of 0.7 to 0.8, indicating fair diagnostic accuracy suitable for screening applications.

### 3.4. Evidence Synthesis

#### 3.4.1. Mechanistic Insights

##### Immune System Modulation

Multiple studies demonstrated significant immune system alterations mediated by gut microbiota in CNS tumor contexts. Kim et al. revealed that brain tumor-induced gut microbiota dysbiosis modulates immunotherapy efficacy through enhanced T-cell circulation, with tryptophan supplementation reversing microbial changes and improving survival outcomes in glioma-bearing mouse models [23].

Green et al. employed single-cell RNA sequencing to demonstrate how human gut microbiota influences immune cell composition and gene expression in the glioma tumor microenvironment [24]. The study revealed differential responses to anti-PD-1 therapy based on microbial composition, with certain bacterial species enhancing therapeutic efficacy through improved T-cell activation and infiltration [24].

Dees et al. established that human gut microbial communities dictate anti-PD-1 therapy efficacy in humanized microbiome mouse models, with responder lines showing high abundances of Bacteroides cellulosilyticus [25]. This groundbreaking study utilized five independent humanized mouse lines derived from healthy human donors, revealing significant differences in immunotherapy response based on microbiome composition [25].

##### Metabolic Pathway Alterations

Short-chain fatty acids emerged as critical mediators of microbiome-CNS tumor interactions. Zhou et al. demonstrated that SCFAs reverse gut microbiota dysbiosis-promoted glioblastoma progression by upregulating M1 macrophage polarization in the tumor microenvironment [26]. The study showed that SCFA supplementation significantly reduced tumor growth and improved survival in preclinical models [26].

Ashwini et al. provided molecular evidence that butyrate inhibits glioblastoma cell proliferation through histone deacetylase-3 (HDAC3) inhibition, suggesting epigenetic mechanisms underlying microbiome-mediated tumor suppression [27].

##### Inflammatory Response Modifications

D’Alessandro et al. demonstrated that gut microbiota alterations affect glioma growth and innate immune cell infiltration in murine models [28]. Antibiotic-induced microbiome depletion led to increased tumor growth and altered immune cell composition, suggesting protective roles of commensal bacteria against tumor development through inflammatory pathway modulation [28].

#### 3.4.2. Clinical Correlations

##### Microbiome Composition in CNS Tumor Patients

Li et al. conducted the most comprehensive clinical analysis, examining 158 participants including 101 brain tumor patients and 57 healthy controls [19]. The study revealed significantly reduced microbial ecosystem richness and evenness in brain tumor patients, with increased abundances of pathogenic bacteria including Fusobacteriota and Proteobacteria, and reduced probiotic bacteria such as *Bifidobacterium* and *Lachnospira* [19]. More pronounced changes were observed in malignant versus benign tumors [19].

Jiang et al. performed the first comparative study of benign and malignant brain tumors, analyzing 32 meningioma patients, 27 glioma patients, and 41 healthy controls [20]. Distinct microbial signatures were identified, with meningioma patients showing increased Enterobacteriaceae, while glioma patients had elevated Fusobacterium and Akkermansia [20]. Both groups lacked short-chain fatty acid-producing probiotics compared to controls [20].

##### Pediatric CNS Tumors

Dossena et al. examined gut microbiota composition in pediatric CNS tumor patients, revealing distinct alterations compared to healthy children [21]. The study identified age-specific microbial changes and suggested that early-life microbiome disruption may influence CNS tumor development and treatment response [21].

#### 3.4.3. Therapeutic Interventions

##### Immunotherapy Enhancement

Weathers et al. analyzed long-term survivors of anti-PD-L1 treated glioblastoma patients, identifying distinct immune, mutation, and gut microbiome features associated with improved outcomes [29]. Patients with favorable microbiome profiles showed enhanced immune activation and prolonged survival, suggesting microbiome-based stratification for immunotherapy selection [29].

Hou et al. demonstrated that gut microbiota mediated individualized efficacy of temozolomide through immunomodulation in glioma patients [30]. The study revealed that specific bacterial species influence drug metabolism and immune response, providing rationale for personalized treatment approaches based on microbiome profiling [30].

##### Microbiome-Targeted Interventions

Several studies explored direct microbiome interventions. Zhou et al. showed that short-chain fatty acid supplementation reversed dysbiosis-promoted glioblastoma progression, while Kim et al. demonstrated that tryptophan supplementation could restore beneficial microbial communities and improve treatment outcomes [23,26].

#### 3.4.4. Clinical Study Results

##### Preclinical Findings

Preclinical studies using mouse models provided mechanistic insights into microbiome-CNS tumor interactions. Kim et al. demonstrated that brain tumor presence induces gut microbiota dysbiosis, which subsequently modulates immunotherapy efficacy through enhanced T-cell circulation in conventional mice [23]. D’Alessandro et al. showed that antibiotic-induced microbiome depletion in conventional mice led to increased glioma growth and altered immune cell infiltration, suggesting protective roles of commensal bacteria [28].

Studies using humanized microbiome models revealed critical translational insights. Dees et al. utilized humanized microbiome mouse models derived from five independent healthy human donors, demonstrating that human gut microbial communities dictate anti-PD-1 therapy efficacy, with responder lines showing high abundances of Bacteroides cellulosilyticus [25]. This approach addresses the translational limitation of conventional mouse microbiomes that differ significantly from human microbiomes.

##### Primary Outcome—Microbial Diversity Analysis

Meta-analysis of clinical studies revealed consistent Shannon diversity reduction across all studies (Table 1, Figure 2). The pooled analysis demonstrated a significant reduction in microbial diversity with Cohen’s d = −1.237, 95% confidence interval from −1.614 to −0.860, and 95% PI from −2.48 to −0.12. This represents approximately 30–40% loss in species richness in brain tumor patients compared to healthy controls.

Heterogeneity assessment revealed moderate between-study variability (I^2^ = 60.5%, τ^2^ = 0.089), which justified the use of random effects modeling.

##### Secondary Outcomes—Bacterial Abundance Meta-Analysis

Analysis revealed significant changes in specific bacterial taxa (Table 3 and Table 4). Potentially pathogenic bacteria showed increases: *Akkermansia muciniphila* (2.23-fold increase) and *Fusobacterium* spp. (2.04-fold increase). Potentially beneficial bacteria showed decreases: *Bifidobacterium* spp. (47% reduction) and *Lachnospira* spp. (56% reduction). We acknowledge that pathogenic vs. beneficial classification at the genus level represents a limitation, as bacterial function varies at the strain level, and some Bifidobacterium strains may act as opportunistic pathogens under specific conditions. Collectively, the integrated synthesis of preclinical and clinical evidence indicates that gut microbiota alterations are consistently associated with CNS tumor biology, despite methodological diversity across studies.

### 3.5. Quality Assessment and Publication Bias

GRADE evidence quality assessment revealed several considerations across multiple domains (Table 6).

Study design was classified as observational with a downgrade of one level due to non-randomized studies. Risk of bias was assessed as moderate with no downgrade due to adequate control groups and standardized methods. Inconsistency was moderate with an I^2^ of 60.5%, resulting in a downgrade of one level due to heterogeneity in methods and populations. Indirectness was assessed as direct with no downgrade due to relevant populations and outcomes. Imprecision was high with a downgrade of one level due to wide confidence intervals and small studies. Publication bias was considered unlikely with no downgrade as there was no statistical evidence. The overall evidence quality was rated as low using the GRADE system.

Publication bias analysis using Egger’s test revealed an intercept of −0.718 with standard error of 0.212 and *p* = 0.627, indicating no significant evidence of publication bias (Table 7).

The funnel plot demonstrated symmetric distribution around the pooled effect, supporting the absence of publication bias.

### 3.6. Sensitivity and Robustness Analysis

Leave-one-out analysis demonstrated robust results across all sensitivity analyses (Table 8).

When excluding Li et al., the pooled effect size was −1.039 with 95% confidence interval from −1.331 to −0.748, showing reduced magnitude but remaining significant [19]. Excluding Jiang et al. resulted in a pooled effect size of −1.421 with confidence interval from −1.732 to −1.110, showing increased magnitude while remaining significant [20]. Excluding Dossena et al. yielded a pooled effect size of −1.221 with confidence interval from −1.738 to −0.703, demonstrating minimal change while remaining significant [21]. Excluding Patrizz et al. produced a pooled effect size of −1.263 with confidence interval from −1.743 to −0.783, showing minimal change while remaining significant [22].

All sensitivity analyses maintained statistical significance with *p* < 0.001 and large effect sizes, confirming the robustness of findings across different combinations of included studies.

## 4. Discussion

This systematic review and meta-analysis provides preliminary evidence for bidirectional microbiome-CNS tumor interactions, with a large pooled effect size for Shannon diversity reduction (Cohen’s d = −1.237). While the findings should be interpreted with caution, they collectively support the concept of a gut–brain–tumor axis that integrates immune, metabolic, and inflammatory signaling pathways. Nevertheless, several methodological and clinical limitations constrain the confidence and translational applicability of these results.

It is important to emphasize that this analysis does not constitute a reprocessing-based meta-analysis of raw 16S rRNA data but rather a quantitative synthesis of published statistical outcomes. This “reported-data meta-analysis” approach has been widely used across biomedical and microbiome studies when raw data harmonization is not feasible, allowing estimation of pooled effects despite inter-study pipeline differences.

The analysis revealed consistent patterns of dysbiosis characterized by reduced diversity with large effect size indicating clinically meaningful disruption, increased pathogenic bacteria including *Akkermansia muciniphila* with 2.23-fold increase and *Fusobacterium* species with 2.04-fold increase, decreased beneficial bacteria including *Bifidobacterium* species with 47% reduction and *Lachnospira* species with 56% reduction, and fair diagnostic performance with pooled AUC of 0.786 suitable for screening applications. The observed microbiome alterations may result from tumor-induced systemic changes affecting gut physiology, or alternatively, pre-existing dysbiosis may influence CNS tumor development through immune modulation that penetrates the altered blood-brain barrier in tumor regions.

The mechanistic framework involves multiple pathways operating within the compromised CNS immune privilege environment characteristic of brain tumors. Tumor-associated inflammation and blood-brain barrier disruption create opportunities for peripheral immune signals, influenced by gut microbiota, to access the CNS compartment. This represents a departure from normal CNS immune privilege, where the blood-brain barrier and specialized immune surveillance limit peripheral immune system access.

The molecular mechanisms underlying microbiome-CNS tumor interactions involve three primary pathways. Immune system modulation occurs through several pathways. Gut microbiota influence systemic T-cell populations that can access brain tumor sites through compromised barriers. The identification of *Bacteroides cellulosilyticus* as predictive of anti-PD-1 response in humanized mouse models suggests specific bacterial species may enhance therapeutic efficacy through improved T-cell activation and CNS infiltration.

Metabolic pathway alterations involve short-chain fatty acid production by beneficial bacteria. SCFA depletion in brain tumor patients may reduce anti-inflammatory signals and impair M1 macrophage polarization in tumor microenvironments. Butyrate specifically demonstrates direct anti-tumor effects through histone deacetylase inhibition, suggesting therapeutic potential for SCFA supplementation.

Inflammatory response modifications demonstrate that commensal bacteria appear to provide protective effects against tumor development through inflammatory pathway modulation, with antibiotic-induced depletion leading to increased tumor growth.

Several evidence-based therapeutic strategies emerge from this analysis. Probiotic supplementation involves targeted administration of potentially beneficial bacteria such as *Bifidobacterium* and *Lachnospira* to restore microbial balance. Prebiotic interventions include dietary modifications promoting beneficial microbial growth. Metabolite supplementation encompasses direct administration of short-chain fatty acids and tryptophan. Combination therapies integrate microbiome interventions with conventional treatments, particularly immunotherapy. Personalized approaches utilize microbiome-based treatment selection algorithms.

This meta-analysis provides the first quantitative synthesis of microbiome-CNS tumor relationships, revealing a larger effect size than reported in individual studies. The pooled AUC of 0.786 represents better diagnostic performance than many traditional biomarkers, supporting clinical utility. The consistency of findings across different populations and methodologies strengthens confidence in the gut-brain-tumor axis concept.

### 4.1. Clinical Translation Challenges

The clinical implications of these findings remain uncertain due to several critical limitations. Our meta-analysis was based on reported outcomes rather than unified raw data analysis, introducing potential bias from heterogeneous analytical approaches across studies. Future research should employ standardized pipelines for raw 16S rRNA data analysis, include multiple diversity indices, and incorporate batch effect correction methods such as MMUPHin.

The bidirectional nature of gut-brain interactions necessitates longitudinal studies to establish causality and temporal relationships. Current cross-sectional designs cannot determine whether microbiome alterations precede tumor development or result from tumor-induced systemic changes.

### 4.2. Limitations

Several study limitations must be acknowledged that impact the strength and generalizability of current evidence. The most significant limitation is the severely restricted sample size across included studies, with only 387 total participants across 6 clinical studies. This evidence base is insufficient for robust clinical conclusions, as individual studies with participant numbers ranging from just 44 to 158 individuals per study were underpowered to detect moderate effects and fall well below what is typically needed to draw robust, definitive conclusions about microbiome changes in brain tumor patients. Small studies are particularly vulnerable to chance findings and may not capture the true diversity of microbiome patterns across different patient populations.

We observed substantial variation in how different research teams conducted their microbiome analyses, resulting in moderate between-study heterogeneity with I^2^ = 60.5%. This methodological heterogeneity encompassed significant differences in DNA extraction protocols, sequencing platforms used to analyze bacterial communities, target regions, bioinformatics pipelines, normalization methods, and the computational pipelines employed to process and interpret the resulting data. Such methodological variations make it challenging to directly compare results across studies, may contribute to conflicting findings in the literature. However, the use of standardized effect size metrics and random-effects modeling in our analysis provides a statistically valid approach for integrating such heterogeneous results, consistent with established methodologies in evidence synthesis when raw sequencing data are unavailable.

The predominance of cross-sectional studies, which comprised 67% of the included studies and employed designs that capture microbiome data at only a single time point, precludes causal inference about the relationship between microbiome alterations and brain tumors. This approach fundamentally prevents us from establishing causal relationships between microbiome changes and brain tumor development or progression, leaving critical questions unanswered about whether microbiome changes precede tumor development, whether alterations are consequences of tumor presence or treatment, and what the temporal relationship is between dysbiosis and disease progression. Longitudinal, prospective studies incorporating repeated sampling and multi-omics profiling are therefore essential to determine whether dysbiosis precedes tumor onset, results from tumor presence, or is modulated by treatment.

Our analysis revealed significant gaps in controlling for key variables that are known to substantially impact the microbiome, representing a critical methodological flaw. The most concerning oversight involved antibiotic use, with only 3 out of 12 studies (25%) controlling for antibiotic exposure while 9 studies (75%) failed to account for this critical factor. This represents a significant limitation given that antibiotics are known to dramatically alter microbiome composition and could have a high impact on study results. Similarly problematic was the near-universal failure to control for dietary factors, with just 1 out of 12 studies (8%) considering dietary influences while 11 studies (92%) proceeded without dietary controls. Given that diet is one of the strongest predictors of microbiome composition, this lack of control represents another high-impact limitation.

Several other moderately impactful factors were also inadequately controlled across the studies we examined. Chemotherapy status was only controlled in 4 studies (33%), meaning that 8 studies (67%) did not account for the effects of cancer treatments on microbiome composition. The presence of comorbidities was controlled in just 2 studies (17%), while 10 studies (83%) did not consider how other health conditions might influence their findings. Finally, non-chemotherapy medications were controlled in only 2 studies (17%), with 10 studies (83%) not accounting for the potential microbiome effects of other drugs their participants might have been taking. This widespread failure to control for major confounding variables significantly limits our ability to draw reliable conclusions about the relationship between brain tumors and microbiome changes and may lead to overestimation of microbiome-tumor associations.

In addition, this meta-analysis was conducted on reported statistical outcomes rather than raw sequencing data, which may have introduced analytical heterogeneity and reduced reliability of pooled estimates.

The included studies show limited geographic and demographic diversity that raises important questions about global applicability. Our analysis revealed a concerning geographic bias, with Asian populations comprising 50% of all study participants, while other populations were predominantly North American and European with limited pediatric representation from only 1 study and inadequately characterized socioeconomic factors across studies. This concentration in specific geographic regions limits our ability to understand how microbiome-brain tumor relationships might vary across different ethnic backgrounds, dietary patterns, environmental exposures, and healthcare systems found globally. The findings may not adequately represent microbiome patterns in European, African, or American populations. Since microbiome composition varies substantially across geographic regions, ethnicities, and dietary patterns, the limited population diversity raises important questions about the global applicability of findings and undermines the strength of evidence supporting a direct relationship between brain tumors and microbiome alterations.

## 5. Conclusions

This systematic review and meta-analysis provides evidence that gut microbiota are significantly altered in CNS tumor patients, with our analysis demonstrating a large pooled effect size of Cohen’s d = −1.237 representing clinically meaningful microbiome disruption. The analysis demonstrates fair diagnostic performance with AUC = 0.786 for microbiome-based biomarkers, supporting their potential clinical utility. However, the evidence quality is low due to small sample sizes, methodological heterogeneity, and study design limitations that significantly impact our ability to draw definitive conclusions.

The key findings reveal statistical evidence for substantial microbiome alterations in CNS tumor patients, though the clinical relevance remains uncertain due to methodological limitations and inadequate control for confounding variables. While microbiome profiling shows promise as a non-invasive biomarker for CNS tumors with fair diagnostic performance, this level of performance is insufficient for immediate clinical implementation. The biological rationale for microbiome-CNS tumor relationships exists through immune, metabolic, and inflammatory mechanisms that provide rational therapeutic targets, but these mechanistic pathways require extensive validation in preclinical models before clinical application.

Some critical limitations significantly constrain the interpretation of current findings. The sample size inadequacy, with only 387 total participants across 6 studies, renders the current evidence base too small for robust clinical conclusions. Methodological heterogeneity across studies, including variable DNA extraction protocols, sequencing platforms, and analysis pipelines, compromises result reliability and limits the validity of pooled analyses. The predominance of cross-sectional study designs prevents determination of causality, leaving fundamental questions unanswered about whether microbiome changes precede tumor development or result from tumor presence and treatment. Additionally, inadequate control for major confounding variables such as antibiotic use, dietary factors, and cancer treatments may lead to overestimation of microbiome-tumor associations.

Although individual studies exhibited methodological limitations, the consistent findings observed across diverse populations and study designs, supported by robust sensitivity analyses, lend credibility to the reported results. Nevertheless, considerable additional research is necessary before clinical translation becomes feasible. The field urgently needs large-scale validation studies employing unified analytical pipelines, with each study requiring a minimum of 1000 participants.

To improve data quality and comparability, existing raw 16S rRNA sequencing datasets should undergo re-analysis using standardized workflows that minimize batch effects. Future diversity analyses must incorporate multiple alpha diversity indices alongside comprehensive beta diversity measures. To establish causality and enhance translational relevance, mechanistic investigations should emphasize gnotobiotic and humanized microbiome mouse models. Finally, longitudinal clinical studies are critical for elucidating the temporal relationships between microbiome alterations and tumor progression.

While the present synthesis is limited by its reliance on reported statistical outcomes rather than harmonized raw 16S rRNA data, it nonetheless provides an essential quantitative baseline to guide future meta-omics efforts conducted under unified analytical frameworks. The microbiome-CNS tumor field shows promise with evidence supporting potential diagnostic utility and therapeutic targets through microbiome-targeted interventions as adjuvants to conventional therapy. Microbiome composition may eventually guide treatment selection, particularly for immunotherapy approaches, as part of personalized medicine strategies. However, the integration of microbiome science with conventional CNS tumor treatment, while representing a promising frontier for improving patient outcomes, requires extensive additional research including mechanistic clarification and rigorous testing of microbiome-targeted interventions.

## Figures and Tables

**Table 1:** Shannon Diversity Index Comparisons (CNS tumor patients vs. healthy controls).

Study	Sample Size	Effect Size (Cohen’s d)	95% CI	Interpretation
Li et al., 2022 [19]	*n* = 158 (101 patients, 57 controls)	−1.634	[−2.005, −1.262]	Large effect
Jiang et al., 2022 [20]	*n* = 100 (59 patients, 41 controls)	−0.866	[−1.282, −0.450]	Large effect
Dossena et al., 2025 [21]	*n* = 65 (32 patients, 33 controls)	−1.269	[−1.802, −0.736]	Large effect
Patrizz et al., 2020 [22]	*n* = 44 (24 patients, 20 controls)	−1.117	[−1.754, −0.479]	Large effect

**Table 2:** Heterogeneity statistics.

Statistic	Value	df	*p*-Value	Interpretation
Q-statistic	7.588	3	0.055	Moderate heterogeneity
I^2^	60.5%	-	-	Moderate between-study variance
τ^2^ (tau-squared)	0.089	-	-	Random effects variance
H^2^	2.53	-	-	Ratio of total to sampling variance

**Table 3:** Potentially pathogenic bacteria (increased in CNS tumor patients).

Bacterial Taxa	Fold Change	95% CI	Studies (*n*)	Clinical Relevance
*Akkermansia* *muciniphila*	2.23×	[1.59, 3.14]	3	Mucin degradation, barrier dysfunction
*Fusobacterium* spp.	2.04×	[1.36, 3.07]	2	Pro-inflammatory, oncogenic potential

**Table 4:** Potentially pathogenic bacteria (decreased in CNS tumor patients).

Bacterial Taxa	Fold Change	95% CI	Studies (*n*)	Clinical Relevance
*Bifidobacterium* spp.	0.53× (47% reduction)	[0.41, 0.67]	4	SCFA production, immune modulation
*Lachnospira* spp.	0.44× (56% reduction)	[0.27, 0.70]	2	Butyrate production, anti- inflammatory

**Table 5:** Diagnostic biomarker performance—individual study performance.

Study	Sample Size	AUC	95% CI	Sensitivity	Specificity	Biomarker Panel
Li et al., 2022 [19]	158	0.770	[0.682, 0.858]	72%	75%	Multi-taxa Classifier
Jiang et al., 2022 [20]	100	0.820	[0.746, 0.894]	85%	78%	6-taxa panel
Dossena et al., 2025 [21]	65	0.740	[0.638, 0.842	68%	71%	Diversity-based

**Table 6:** GRADE evidence profile.

Outcome	Studies (*n*)	Participants (*n*)	Effect Size	95% CI	Quality Assessment	Overall Quality
Shannon Diversity	4	367	−1.237	[−1.614, −0.860]	⊕⊕◯◯	low
Diagnostic AUC	3	323	0.786	[0.781, 0.791]	⊕⊕◯◯	low
Bacterial Abundance	4	367	Variable	See Table S5	⊕◯◯◯	very low

**Table 7:** Publication bias assessment.

Test	Statistic	SE	t-Value	df	*p*-Value	95% CI Lower	95% CI Upper	Interpretation
Egger’s Test	−0.718	0.212	−3.387	2	0.627	−1.631	0.195	No significant bias
Begg’s Test	τ = −0.333	-	z = −0.632	-	0.527	-	-	No significant bias

**Table 8:** Sensitivity analysis results.

Analysis Type	Pooled Effect	95% CI Lower	95% CI Upper	I^2^	Q-Statistic	*p*-Value
All studies	−1.237	−1.614	−0.860	60.5%	7.588	<0.001
Exclude Li et al. [19]	−1.039	−1.331	−0.748	31.2%	2.906	<0.001
Exclude Jiang et al. [20]	−1.421	−1.732	−1.110	48.9%	3.916	<0.001
Exclude Dossena et al. [21]	−1.221	−1.738	−0.703	70.8%	6.849	<0.001
Exclude Patrizz et al. [22]	−1.263	−1.743	−0.783	68.4%	6.329	<0.001
Fixed effects model	−1.298	−1.543	−1.053	-	-	<0.001

## Data Availability

Data will be available as Supplementary Files.

## Supplementary Materials

The following supporting information can be downloaded at: https://www.mdpi.com/article/10.3390/ijms262110721/s1.
