# Supplementary material for: Gut Microbiota and Central Nervous System Tumors: A Comprehensive Systematic Review and Meta-Analysis of Microbiome-CNS Interactions
Source: Int J Mol Sci. 2025 Nov 4;26(21):10721. doi: 10.3390/ijms262110721 (PMC12609029; doi:10.3390/ijms262110721)
Supplement: Supplementary file 1 [file ijms-26-10721-s001.zip › ijms-3863092-supplementary.pdf]

Table S1: Study characteristics and quality assessment.

study	year	design	population	Sample size	age (mean±SD)	Tumor types	Microbiome method	Quality score
Li et al.	2022	Cross-sectional	Chinese adults	158 (101 patients, 57 controls)	52.3±12.8	Mixed CNS tumors	16S rRNA V3-V4	7/9
Jiang et al.	2022	Case-control	Chinese adults	100 (59 patients, 41 controls)	48.7±15.2	Glioma, Meningioma	16S rRNA V4	6/9
Dossena et al.	2025	Cross-sectional	Italian pediatric	65 (32 patients, 33 controls)	8.5±4.2	Pediatric CNS	16S rRNA V3-V4	8/9
Patrizz et al.	2020	Longitudinal	US adults	44 (24 patients, 20 controls)	56.1±14.5	Glioblastoma	16S rRNA V4	7/9

Table S2: Raw data for Shannon Diversity meta-analysis.

study	group	n	mean	SD	SE	95% CI Lower	95% CI Upper
Li et al. 2022	Tumor	101	2.8	0.4	0.040	2.72	2.88
Li et al. 2022	control	57	3.4	0.3	0.040	3.32	3.48
Jiang et al. 2022	Tumor	59	2.9	0.5	0.065	2.77	3.03
Jiang et al. 2022	Control	41	3.3	0.4	0.062	3.18	3.42
Dossena et al. 2025	Tumor	32	2.5	0.6	0.106	2.28	2.72
Dossena et al. 2025	Control	33	3.2	0.5	0.087	3.02	3.38
Patrizz et al. 2020	Tumor	24	2.7	0.4	0.082	2.53	2.87
Patrizz et al. 2020	Control	20	3.1	0.3	0.067	2.96	3.24

Table S3: Effect size calculations.

Study	Cohen's d	SE(d)	Variance	Weight (Fixed)	Weight (Random)	95% CI Lower	95% CI Upper
Li et al. 2022	-1.634	0.189	0.036	27.78	23.45	-2.005	-1.262
Jiang et al. 2022	-0.866	0.212	0.045	22.22	20.87	-1.282	-0.450
Dossena et al. 2025	-1.269	0.272	0.074	13.51	15.32	-1.802	-0.736
Patrizz et al. 2020	-1.117	0.325	0.106	9.43	12.18	-1.754	-0.479
Pooled (Random)	-1.237	0.193	0.037	-	71.82	-1.614	-0.860

Table S4: Subgroup analysis results.

Subgroup	Studies (n)	Pooled Effect Size	95% CI	I^2
**By tumor type**				
Glioblastoma only	2	-1.376	[-1.89, -0.86]	45.2%
Mixed tumors	2	-1.451	[-2.34, -0.56]	72.1%
**By analytical method**				
16S V3-V4 region	2	-1.451	[-1.98, -0.92]	38.7%
16S V4 region	2	-0.992	[-1.65, -0.33]	66.3%
**By Population**				
Adult studies	3	-1.206	[-1.67, -0.74]	58.9%
Pediatric studies	1	-1.269	[-1.80, -0.74]	N/A

Table S5: Bacterial abundance meta-analysis data.

Bacterial Taxa	Studies (n)	Log2 Fold Change	SE	95% CI Lower	95% CI Upper	Fold Change	Direction
Akkermansia muciniphila	3	1.16	0.25	0.67	1.65	2.23x	Increased
Fusobacterium spp.	2	1.03	0.30	0.44	1.62	2.04x	Increased
Bifidobacterium spp.	4	-0.92	0.18	-1.27	-0.57	0.53x	Decreased
Lachnospira spp.	2	-1.20	0.35	-1.89	-0.51	0.44x	Decreased
Enterobacteriaceae	2	0.85	0.28	0.30	1.40	1.80x	Increased
Bacteroides spp.	3	-0.45	0.22	-0.88	-0.02	0.73x	Decreased

Table S6: Diagnostic performance meta-analysis.

Study	TP	FP	FN	TN	Sensitivity	Specificity	PPV	NPV	Accuracy	AUC	AUC SE
Li et al. 2022	73	14	28	43	0.72	0.75	0.84	0.61	0.73	0.770	0.045
Jiang et al. 2022	50	9	9	32	0.85	0.78	0.85	0.78	0.82	0.820	0.038
Dossena et al. 2025	22	10	10	23	0.68	0.71	0.69	0.70	0.69	0.740	0.052
Pooled	145	33	47	98	0.75	0.75	0.81	0.68	0.75	0.786	0.003

Table S7: Clinical significance thresholds.

**Outcome**	**Threshold**	**Observed Value**	**Clinical Significance**	**Interpretation**
Cohen's d	±0.2 (small), ±0.5	-1.237	large	Clinically meaningful
AUC	0.5 (no discrimination), 0.7	0.786	fair	Moderate clinical
I^2	<25% (low), 25-75%	60.5%	Moderate	Acceptable
p-value	<0.05 (significant)	<0.001	Significant	Statistically significant
